# In tendons, differing physiological requirements lead to functionally distinct nanostructures

**DOI:** 10.1038/s41598-018-22741-8

**Published:** 2018-03-13

**Authors:** Andrew S. Quigley, Stéphane Bancelin, Dylan Deska-Gauthier, François Légaré, Laurent Kreplak, Samuel P. Veres

**Affiliations:** 1 Department of Physics and Atmospheric Science, Dalhousie University, Halifax, Canada; 2 Institut National de la Recherche Scientifique, Centre Énergie, Matériaux, Télécommunication, Varennes, Canada; 3 Department of Medical Neuroscience, Dalhousie University, Halifax, Canada; 4 School of Biomedical Engineering, Dalhousie University, Halifax, Canada; 5 Division of Engineering, Saint Mary’s University, Halifax, Canada

## Abstract

The collagen-based tissues of animals are hierarchical structures: even tendon, the simplest collagenous tissue, has seven to eight levels of hierarchy. Tailoring tissue structure to match physiological function can occur at many different levels. We wanted to know if the control of tissue architecture to achieve function extends down to the nanoscale level of the individual, cable-like collagen fibrils. Using tendons from young adult bovine forelimbs, we performed stress-strain experiments on single collagen fibrils extracted from tendons with positional function, and tendons with energy storing function. Collagen fibrils from the two tendon types, which have known differences in intermolecular crosslinking, showed numerous differences in their responses to elongation. Unlike those from positional tendons, fibrils from energy storing tendons showed high strain stiffening and resistance to disruption in both molecular packing and conformation, helping to explain how these high stress tissues withstand millions of loading cycles with little reparative remodeling. Functional differences in load-bearing tissues are accompanied by important differences in nanoscale collagen fibril structure.

## Introduction

While collectively referred to as tendons, the physiological functions served by the tissues that connect muscle to bone vary considerably within certain animals, including humans. Tendons like the digital extensors and flexors of the hand transmit forces in such a way that fingers can be moved with great precision. Other tendons, like the Achilles, function as springs that enable locomotive activities such as running and jumping to be performed efficiently by storing energy during deceleration, and then releasing it to help power acceleration^1–3^.

Tendons that store and release energy face differing functional demands to those that are primarily positional in nature. Unlike positional tendons, energy storing tendons must be able to withstand large forces applied in a highly repetitive manner. When running, for example, tension in the Achilles tendon during ground contact exceeds 12 times body weight^4^; in the case of a marathon, the tendon must endure this loading about 25,000 times without rest. In other tissues such as bone, demanding mechanical loading regimes are dealt with via remodeling, where fatigue damage occurs, but its excessive accumulation is prevented by turnover of the damaged components^5^. Surprisingly, despite undergoing high stress cyclic loading, energy storing tendons do not undergo appreciable remodeling^6,7^. Rather than continually repair accrued damage, these specialized tendons appear to have evolved highly fatigue resistant structures.

With their differing functions in mind, we wanted to determine if energy storing and positional tendons were composed of functionally distinct collagen fibrils. Collagen fibrils are very fine, precision built, biological cables, each millimeters long but only a hundred nanometers or so in diameter^8,9^. Fibrils gain tensile strength via intermolecular crosslinking, which transmits load from one triple-helical collagen molecule to the next within the fibril structure^10–12^. Tendons are composed of millions of these collagen fibrils packed in parallel, which together make up about three-quarters of a young, healthy tendon’s dry weight^13,14^.

**Figure 1:**
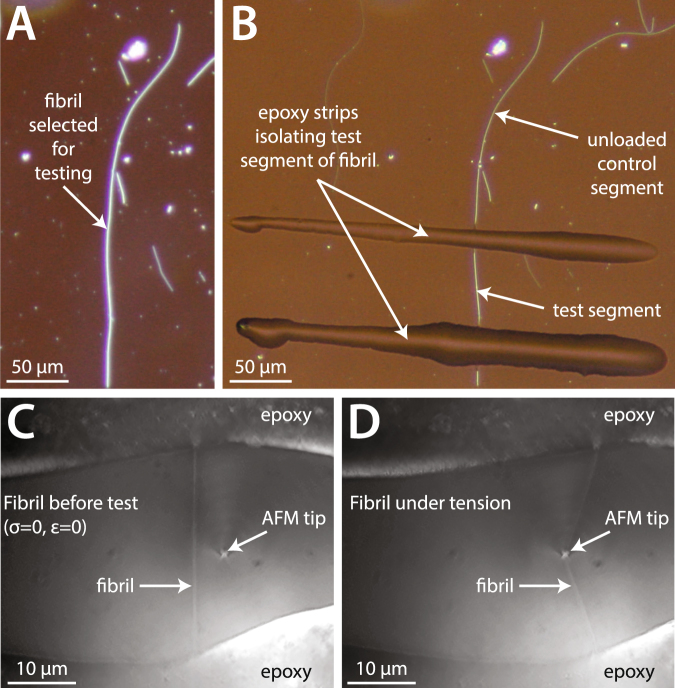
Preparation and bowstring tensile testing of single collagen fibrils conducted using AFM. (**A**) Darkfield microscopy was used to select fibrils for mechanical testing. (**B**) The same fibril shown in (**A**) after preparation for mechanical testing. Epoxy strips were used to isolate a 50-μm-long test segment of the fibril, which would be pulled to rupture using AFM. The portion of each fibril outside the epoxy strips served as an unloaded control. (**C**) Video image before contact of the AFM tip with the fibril. The AFM tip was placed in contact with the glass substrate and held static. (**D**) With the AFM tip held stationary, the stage was moved to the right at 1 μm/s, pulling fibrils into a bowstring geometry until rupture occurred.

We extracted collagen fibrils from two tendons of the bovine forelimb: from the superficial digital flexor (SDF) tendon, an energy storing tendon subjected to a maximum *in vivo* stress of about 70 MPa, and the common digital extensor (CDE) tendon, a positional tendon that experiences a maximum *in vivo* stress of only about 10 MPa^15^. We then performed tension to rupture tests on individual fibrils from both tendon types, using an atomic force microscope (AFM) to load single nanoscale collagen fibrils in a bowstring geometry (Fig. 1)—an approach that has been used previously for testing other thin, extensible biological fibres^16,17^. The responses of the two types of collagen fibrils to loading were investigated through: (i) tensile stress-strain behavior, (ii) high resolution AFM imaging to assess alterations to fibril structure, (iii) polarization-resolved second harmonic generation (SHG) imaging to assess alterations to the alignment of collagen molecules within the fibrils, and (iv) confocal fluorescence microscopy to assess alterations to the triple-helical structure of collagen molecules. We find that the collagen fibrils from these two types of tendon respond differently to applied elongation, with differing mechanisms of extensibility, and differing susceptibilities to structural disruption. Our results provide a new understanding of the structure-function relationships that exist among the nanoscale building blocks of load-bearing collagenous tissues.

## Results

All numerical data are shown as mean ± standard deviation. Quantitative data were first analyzed using a two-way ANOVA with the factors tendon type and animal of origin. For cases where animal of origin was a significant main effect, or where a significant interaction effect existed, data for the two tendon types are shown separated by animal of origin.

### Stress-strain response of single collagen fibrils loaded to rupture

With the bowstring stretching technique, the segment of the collagen fibril undergoing mechanical testing is progressively recruited in tension after initial contact by the AFM tip as it is gradually detached from the underlying glass substrate, as shown previously by Quigley *et al*.^18^. As a result, the stress-strain response of fibrils cannot be accurately determined for the first few percent elongation: from the fibril’s resting state prior to initial AFM tip contact (the zero stress, zero strain condition) to the point at which the full length of the test segment is in tension and stress-strain data can be collected. For this portion of each fibril’s stress-strain curve (from 0 to ~5% strain) the current work used a linear approximation, shown as a dashed line in the presented figures. Previous mechanical testing of hydrated single collagen fibrils has shown that their stress-strain responses are actually concave upward for the first 1–2% strain, but then become linear^19,20^. While an acknowledged limitation of our technique, the inability to capture stress-strain data below ~5% strain does not impact the findings that we present below.

**Figure 2:**
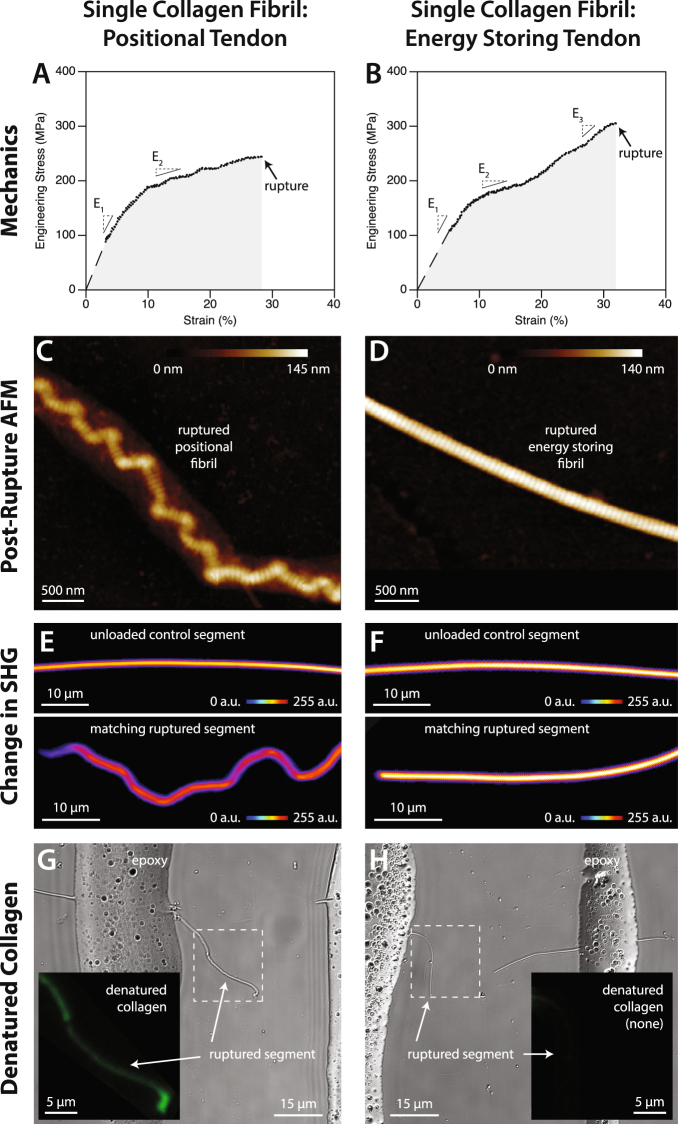
Response of single collagen fibrils from positional tendons (left column) and energy storing tendons (right column) to tensile rupture. (**A**,**B**) Energy storing fibrils stiffen at high strain, while positional fibrils do not. The dashed line indicates the initial portion of the stress-strain curve that could not be captured using the bowstring technique, and was therefore approximated. (**C**,**D**) In response to rupture, positional fibrils undergo a repeating kink distortion (“discrete plasticity”^33,56^) along their entire length. Energy storing fibrils are not disrupted by rupture. (**E**,**F**) Compared to unloaded control segments from the same fibrils, the decrease in SHG intensity shows that rupture causes molecular disorder in positional fibrils, but not in energy storing fibrils. (**G**,**H**) Bright field and confocal fluorescence images (insets) of ruptured collagen fibrils after staining with a fluorescently-labelled collagen hybridizing peptide that binds to denatured collagen, but not native, triple-helical collagen. In addition to disorder, rupture also causes denaturation (uncoiling) of the triple-helical collagen molecules in positional tendons. No denaturation occurs in energy storing fibrils.

Beyond ~5% strain, collagen fibrils from energy storing tendons elongated differently than those from positional tendons (Fig. 2A,B; Supplementary Figs S1, S2). Of the 21 fibrils from positional tendons that were tested, 19 displayed a two-phase load-elongation response when pulled to rupture (Fig. 2A, Fig. S1). At approximately 10% elongation the positional fibrils became easier to extend, with their stiffness decreasing markedly and then remaining relatively constant until abrupt rupture. During the initial phase of extension, from rest to ~10% strain, fibril elongation is likely resisted by entropic mechanisms, where the applied force straightens the triple helix of individual collagen molecules^21,22^. Under sufficient load, molecules then begin to slide longitudinally relative to one another^23^, giving rise to the reduction in fibril modulus that we observed, and as previously predicted^24,25^. In contrast to this, 12 of the 17 fibrils from energy storing tendons that were tested displayed a three-phase load-elongation response (Fig. 2B, Fig. S2). After becoming more compliant with the onset of molecular sliding (again at ~10% elongation), energy storing fibrils became noticeably stiffer at 15% strain, which persisted until abrupt fibril rupture. The differing stress-strain responses that we observed between positional and energy storing fibrils are very similar to data obtained by Svensson *et al*.^19^ using a different AFM-based tensile testing method and different sources of fibrils: human patellar tendon and rat tail tendon. The energy storing bovine SDF fibrils behave as the energy storing human patellar tendon fibrils, while the positional CDE fibrils behave as the positional rat tail tendon fibrils. Interestingly, Liu *et al*.^26^ found that collagen fibrils from rat patellar tendon function similarly to the positional fibrils mentioned above, suggesting that the patellar tendon of rat may not function as an energy storing tendon.

**Figure 3:**
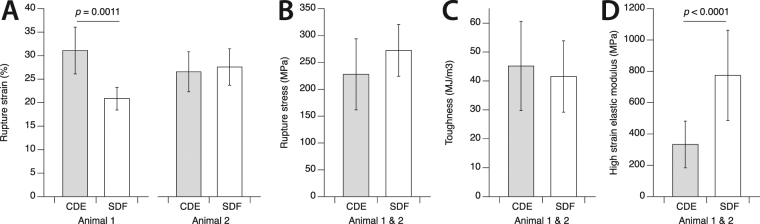
Tensile mechanical properties for single collagen fibrils from positional CDE tendons and energy storing SDF tendons from bovine forelimb. (**A**) For tendons from the first animal, the positional fibrils were significantly more extensible than the energy storing fibrils. The same difference was not observed for the fibrils from the second animal. (**B**,**C**) Positional and energy storing fibrils had statistically similar rupture stress and toughness. (**D**) Consistent with the observed presence of late phase stiffening in energy storing fibrils but not in positional fibrils, the high strain elastic modulus, calculated over the final 10% strain prior to rupture for each fibril, was significantly greater for energy storing fibrils than for positional fibrils. For rupture strain, rupture stress, and toughness, it is important to remember that the values shown here are almost certainly underestimates of the fibrils’ material properties, as stress concentration at points of load application would have caused premature rupture. Note that (**B**,**C**), and (**D**) are calculated based on the cross sectional area of fibrils in the dry condition.

Comparing the mechanical properties of the collagen fibrils tested from positional and energy storing tendons showed only slight differences, with the exception of high strain elastic modulus (Fig. 3). The rupture strain of fibrils depended on animal of origin, where the positional fibrils were significantly more extensible than the energy storing fibrils for one animal, but not the other animal. Energy storing fibrils were ~20% stronger than positional fibrils, but this difference failed to reach significance (*p* = 0.07). Both fibril types were equally resistant to rupture, having toughness values that were similar to one another. For rupture strain, rupture stress, and toughness, it is important to remember that these values are almost certainly underestimates of the fibrils’ material properties, as stress concentration at points of load application would have caused premature rupture. With nearly all positional fibrils having a two-phase stress-strain response (19 of 21), and most energy storing fibrils having a three-phase stress-strain response (12 of 17), a very significant difference in elastic modulus at high strain existed between the two fibril types. When calculated over the final 10% strain prior to rupture, high strain elastic modulus was 774 ± 288 MPa for the energy storing fibrils and 333 ± 149 MPa for the positional fibrils (*p < *0.0001). It should be noted that the mechanical properties given in Fig. 3B–D were calculated based on the dry cross-sectional areas of the tested fibrils, as is common in other single fibril studies^19,20,27–29^. From the dry state, the diameter of collagen fibrils increases by a factor of about 1.9 when hydrated in PBS^30^. Mechanical data for hydrated fibrils is easily calculated from the data presented in Fig. 3B–D by dividing by 3.6.

The presence of late phase stiffening in collagen fibrils from energy storing tendons indicates that, unlike those in positional tendons, extension via molecular sliding is limited, after which additional extension must occur via further molecular straightening and direct stretching of collagen polypeptide α-chains^22^. Collagen molecules making up the common digital extensor tendon of bovine forelimb—the source of the positional tendon fibrils used here—are covalently joined by divalent crosslinks^31^. Collagen crosslinking within the fibrils of energy storing tendons appears to be different: chemical analyses of equine superficial digital flexor tendons show the presence of trivalent crosslinks^32^, consistent with thermal analyses of the bovine tendon equivalent^31^. Our results are consistent with atomistic modeling predictions of how crosslinking chemistry controls the mechanisms of collagen fibril elongation^25^: divalent crosslinks are unable to inhibit molecular sliding, which continues until abrupt fibril rupture, as demonstrated in the positional fibrils. Trivalent crosslinks limit molecular sliding, forcing extension to proceed via the higher stiffness mechanisms of increased molecular straightening and covalent bond stretching, as seen in the energy storing fibrils.

### Ultrastructural changes to collagen fibrils following rupture

By limiting molecular sliding, the trivalent crosslinks present in the collagen fibrils of energy storing tendons may reduce the potential for mechanical disruption of fibril structure, resulting in greater fatigue resistance, and contributing to these tendons’ ability to function with minimal remodeling. To explore whether energy storing fibrils were more resistant to mechanical disruption than positional fibrils, we dehydrated the single fibrils that we had ruptured and then performed high resolution AFM imaging. Unloaded segments of the same fibrils served as controls for comparison.

**Figure 4:**
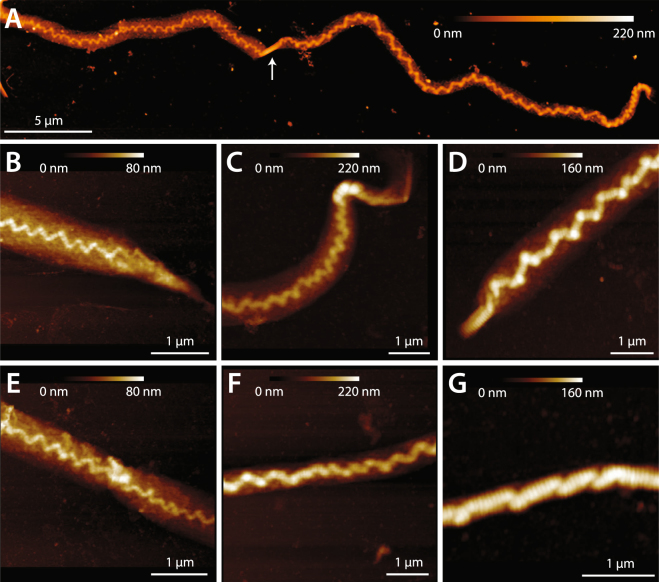
AFM images of dried positional CDE fibrils after rupture. (**A**) Disruption to the structure of positional fibrils extended along the full ~50 μm length of the ruptured segments, with the exception of short, intact “nodes” in some fibrils (arrow). (**B**–**D**) Images from the rupture site. The core of the fibrils is heavily kinked, and surrounded by a shell of highly disordered, denatured collagen. (**E**–**G**) Images from ruptured positional fibrils, distant from the rupture site, showing damage similar to that present in the immediate vicinity of the rupture location (**B**–**D**).

**Figure 5:**
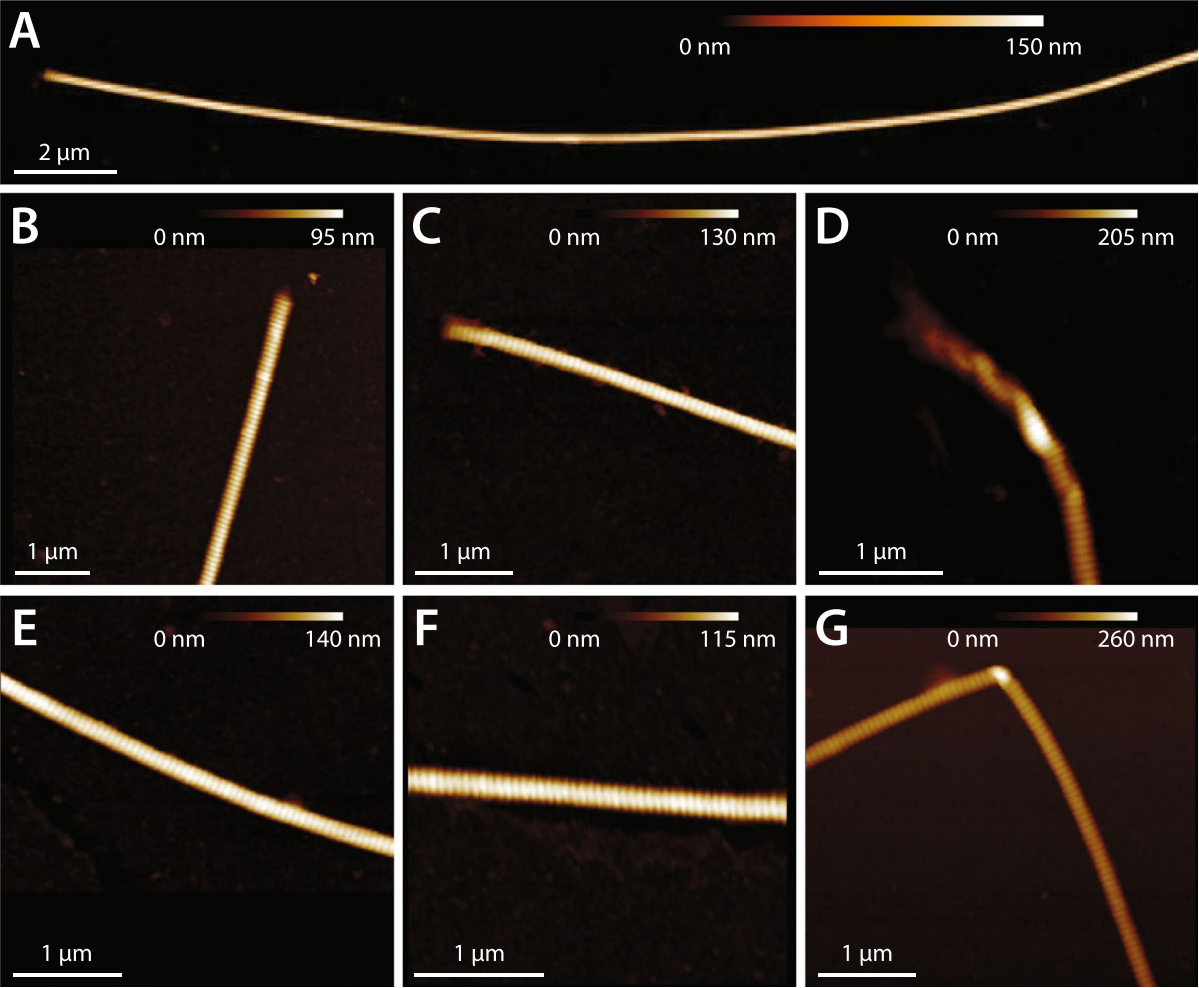
AFM images of dried energy storing SDF fibrils after rupture. (**A**) For most energy storing fibrils, rupture did not cause distortion to the fibril structure along any part of the ~50 μm test segment length. (**B**–**D**) Images from the rupture site. (**B**,**C**) At the rupture site, most fibrils appeared to have cleanly broken, with no distortion or fraying of the fibril structure. (**D**) Occasionally, a small amount of kinking was observed. The periphery of the fibrils always remained intact, however, not undergoing delamination as commonly seen for the positional CDE fibrils (Fig. 4). (**E**–**G**) Images from ruptured energy storing fibrils, distant from the rupture site. (**E**,**F**) Again, most fibrils did not show any ultrastructural changes, appearing straight and D-banded as normal. (**G**) Occasionally, isolated sharp bends were observed.

**Figure 6:**
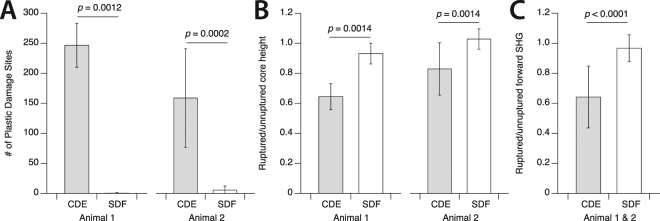
(**A**) In response to rupture, positional fibrils sustained numerous discrete kink-type sites of damage along their length: the average was 205 kinks/50-μm-long test segment (counted from AFM images, as in Fig. 4A). Energy storing fibrils typically did not form any kinks. (**B**) Along with the generation of repeating kinks, positional fibrils lost approximately 25% of their height as material from the fibril periphery delaminated forming a loose shell of denatured collagen surrounding the fibrils. Again, the structure of energy storing fibrils was unaffected by rupture. (**C**) The disruption to ruptured positional fibrils seen in AFM was accompanied by a large reduction in forward SHG signal in polarization-corrected maximum intensity maps, relative to the unloaded control segments of the same fibrils. No significant decrease was observed for energy storing fibrils.

Extending positional collagen fibrils to rupture caused significant disruption to fibril structure over their entire length (Figs 2C and 4). Collagen from the surface of the fibrils delaminated, forming a loose shell that lacked D-banding, indicating a significant level of molecular disorder compared to the native state. Disruption to the inner core of positional fibrils occurred at numerous, discrete locations, resulting in serial kinks that were separated by regions of intact D-banding (Figs 2C and 4G), and hence undisturbed molecular packing. The two damage features noted, a disorganized, delaminated shell and serial kinking, have been previously observed in collagen fibrils from overloaded positional tendons^30,31,33^, indicating that positional fibrils behave similarly when ruptured individually as they do during whole tendon overload. Energy storing fibrils responded very differently to tensile rupture. Unlike positional fibrils, rupture did not cause significant structural disruption to the energy storing fibrils, which retained their native D-banding and uniform linear appearance (Figs 2D and 5). Energy storing fibrils did not readily form kinks in response to rupture (Fig. 6A), and did not experience shell delamination or the associated reduction in fibril core height seen in positional fibrils (Fig. 6B). Again, the structural effects of rupture on the isolated energy storing fibrils seen in the current study is consistent with those seen after rupture of macroscale samples from the same energy storing tendon model^31^, indicating that energy storing fibrils also behave similarly when ruptured individually as they do during whole tendon overload.

### Molecular-level changes to collagen fibrils following rupture

To provide quantitative measures of the molecular-level changes caused by rupture within both fibril types, we performed SHG imaging of the single, ruptured fibrils. Due to the coherent nature of the SHG process, the signal depends not only on the number of triple helices within the focal volume, but also on their organization. SHG imaging showed that rupture caused significant molecular-level damage within positional fibrils: compared to unloaded segments from the same fibrils, rupture caused a 30% reduction in SHG signal in maximum forward scattered intensity projection images (Figs 2E and 6C). For energy storing fibrils, the corresponding reduction was only 2% (Figs 2F and 6C), indicating that extension to rupture had minimal impact on molecular structure, consistent with their normal appearance under AFM imaging (Fig. 2D).

**Figure 7:**
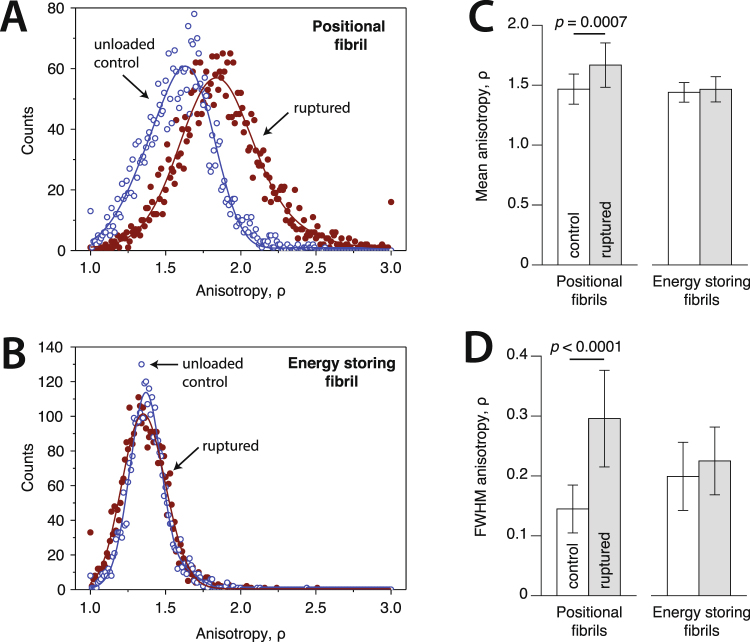
Anisotropy of the second harmonic generation signal for ruptured energy storing and positional fibrils compared to unloaded control segments from those same fibrils. (**A**,**B**) Anisotropy measurements were made at multiple locations along the length of the unloaded control and ruptured segments of each fibril. Values for a representative positional (**A**) and energy storing (**B**) fibril are shown. (**C**,**D**) Rupture only affected the anisotropy parameter for positional fibrils, most notably more than doubling the level of molecular heterogeneity (**D**).

The use of polarization-resolved SHG allowed us to further probe how the molecular structure of collagen fibrils was affected by rupture via measurement of the anisotropy parameter ρ for both the ruptured and unloaded fibril segments. Anisotropy ρ is calculated as the ratio of the second-order nonlinear optical susceptibility tensor components parallel and perpendicular to the fibril axis^34,35^, and has been show to reflect the degree of molecular alignment within each pixel^34,36^. When calculated at multiple locations along the length of a single fibril (Fig. 7A,B), the mean value of anisotropy ρ provides information on the average structure of collagen molecules within a fibril (both alignment and triple-helix conformation), while the dispersion of ρ values indicates the level of homogeneity in molecular structure with length. Anisotropy values were calculated using 200 nm pixels over the entire ruptured and unruptured fibril segments. For a perfectly isotropic arrangement of collagen molecules, ρ = 1. Values of ρ > 1 indicate increased molecular alignment. For positional fibrils, overload increased mean anisotropy (Fig. 7C), indicating that the molecules that had retained their triple-helical conformation following fibril rupture were, on average, better aligned with the fibril axis than they were before loading. Our interpretation is that those molecules making up the core of positional fibrils were pulled into greater alignment by the applied load, while those forming the fibril’s periphery, which may have had lower ρ values to start with (being less well aligned with the fibril axis in the unloaded state)^37,38^, were denatured, consequently disappearing from the anisotropy data following rupture (evidence that molecular denaturation did indeed occur is presented in the next paragraph). In addition to altering average molecular alignment, rupture also significantly increased the dispersion of ρ values for the positional fibrils (Fig. 7D). Compared to their unloaded structure, this indicates that rupture led to greater inconsistency in molecular alignment with length, consistent with the repeating kink deformations visible in the core of the ruptured positional fibrils (Fig. 4). For energy storing fibrils, neither mean anisotropy nor peak width changed significantly (Fig. 7B–D), again indicating that rupture caused little disruption to molecular structure.

**Figure 8:**
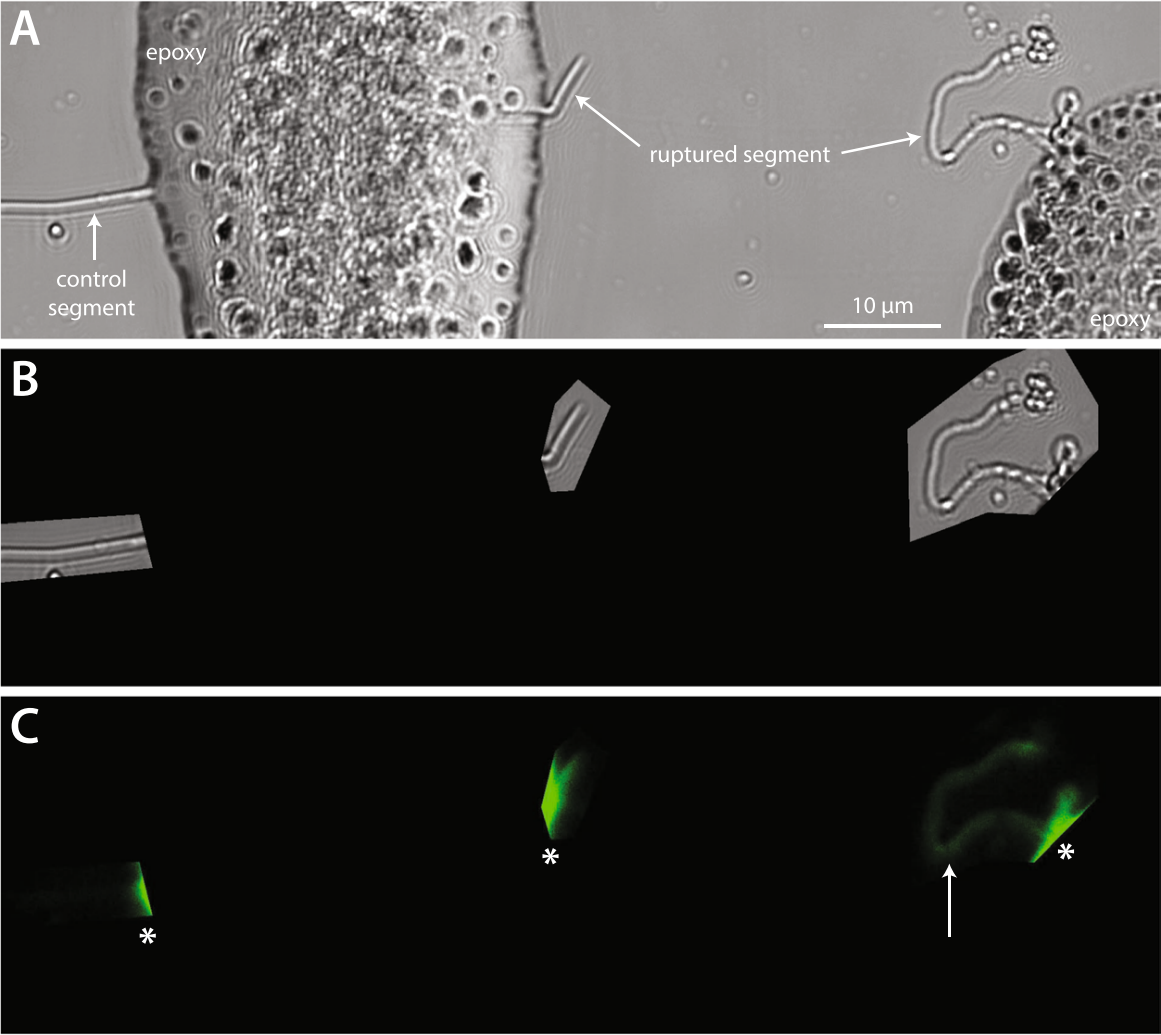
Fluorescent imaging of denatured collagen in a ruptured positional CDE fibril. (**A**) Brightfield image showing both unloaded control and ruptured segments of the fibril. (**B**) Defined regions of interest for fluorescent imaging. (**C**) The right-hand portion of the ruptured segment stains positively for denatured collagen (arrow), while the control segment shows no evidence of denatured collagen. The intense fluorescent signals (*) originate from the epoxy at the edges of the scanned regions of interest.

**Figure 9:**
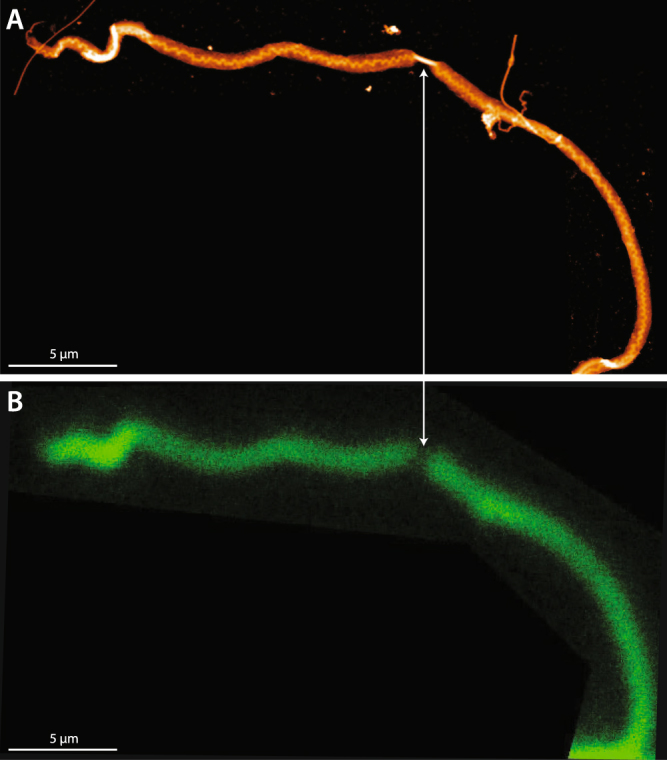
Correspondence between positional fibril disruption due to rupture as seen in dry AFM imaging (**A**) and collagen denaturation as visualized via a fluorescently-labelled collagen hybridizing peptide (**B**). (**A**) Some ruptured positional fibrils contained short undamaged “nodes” (arrow). (**B**) As expected, the undamaged node shows no indication of denatured collagen. The rest of the ruptured segment showing kinking and periphery delamination in AFM stains positively for the presence of denatured collagen.

Earlier research has indicated that overload may cause irrecoverable unwinding of the collagen triple-helix, often termed molecular denaturation^39^. While SHG is sensitive to the molecular structure of collagen fibrils, it is unable to discriminate between changes in molecular alignment and molecular denaturation. To determine if the observed structural changes to positional fibrils included molecular denaturation, we stained the single ruptured fibrils with a fluorescently-labeled collagen hybridizing peptide (CHP) that binds to denatured collagen, but not native collagen^40,41^. For the unloaded, control segments of fibrils, neither positional nor energy storing fibrils showed evidence of denatured collagen. Extending positional fibrils to rupture caused significant levels of molecular denaturation (Figs 2G, 8 and 9). In contrast, the triple helical structure of collagen molecules within energy storing fibrils was unaffected by fibril rupture (Fig. 2H).

## Discussion

During preparation for mechanical testing, fibrils were dried and then rehydrated in 1 × PBS. Tendon-level study has shown that long-term dry storage over months increases tendon strength and stiffness^42^. We do not expect the dry storage that we used (maximum of four days prior to rehydration) to have significantly impacted fibril mechanics. Rehydration and testing was done in 1 × PBS to mimic *in vivo* osmotic conditions. It is interesting to note that neither collagen fibril nor tendon fascicle mechanics appear to vary significantly with salt concentration of the testing medium^27^.

Conducting tension-to-failure testing of single collagen fibrils is experimentally challenging due to their nanoscale diameter and high extensibility. The bowstring technique is a good tool for testing materials with these characteristics, and has indeed been used previously to study the tensile properties of other highly extensible, small diameter biological fibres, including fibrin^16^ and fibronectin^17^. Despite its utility, the bowstring technique is not without drawbacks. As mentioned previously, stress-strain data cannot be captured for the first few percent strain while the fibril is detached from the glass substrate. Though a limitation of the technique, this did not impact our present findings. In the bowstring geometry, although the majority of the fibril being tested is in tension, at the support and loading points a complex state of normal and shear stresses would exist, leading to higher localized strains and causing early failure. This is not unique to the bowstring technique, however; the commonly used vertical AFM pulling method for testing single fibrils is subject to the same issue^19,20,27,28,43,44^. In this study, as well as in those previously mentioned, the measured values of ultimate stress, ultimate strain, and toughness would certainly be underestimates of the true values. The value of the high strain modulus that we have presented should also be viewed as an approximation. While higher local strains at the loading points in our experiments would have been present, two important points should be noted. First, structural damage to the ruptured positional fibrils extended along the full length of the test segments (Figs 4A and 9), indicating that the entire test segment experienced a very high level of applied strain. The form of longitudinally repeating kink damage seen—discrete plasticity—only occurs to collagen fibrils when tendons are extended beyond their yield point^33^. Second, despite some level of uncertainty regarding the true value of high strain modulus, the load-elongation responses of the positional and energy storing fibrils that we tested were certainly distinct.

In terms of sample numbers, other studies that have performed tension-to-rupture testing on single hydrated collagen fibrils have tested 12 fibrils total^26^, 25 fibrils total^19^, and 26 fibrils total^29^. The current study tested a total of 38 fibrils, substantially more. There is significant uncertainty regarding the levels of structural and mechanical variability that exist between different fibrils from a single tendon. For this reason, we chose to test more fibrils from fewer animals rather than the converse. Our results, however, indicate that some inter-animal differences in fibril mechanics do exist. Although an experimental challenge, future studies should simply test more fibrils: tendons from several individuals should be used, and several fibrils from each tendon should be tested.

## Conclusion

Collagen fibrils from energy storing tendons are clearly structurally and mechanically different to those making up positional tendons. The presence of trivalent crosslinks within energy storing fibrils appears to limit molecular sliding, making the fibril structure resistant to permanent and irrecoverable unwinding of the collagen triple-helix. The ability to retain normal structure under severe loading conditions may explain the ability of energy storing tendons to function with little reparative cellular remodeling during a person’s lifetime. An open question remains: what advantage do positional tendons gain by having collagen fibrils that are susceptible to significant structural disruption when overloaded? The presence of trivalent crosslinking has been linked to a decreased enzymatic susceptibility of collagen fibrils^45^. It may be that frequent turnover of collagen fibrils in positional tendons, and thus limitation of advanced glycation endproduct crosslink accumulation^46^, is important to the tendons’ proper physiologic function, and that susceptibility to severe mechanical disruption is a necessary trade-off for a fibrillar structure that facilitates remodeling.

## Methods

### Tendon samples

One forelimb was harvested from each of two 24–36 month-old steers killed for food at a local abattoir (Oulton’s Farm, Nova Scotia, Canada). The forelimbs were transferred on ice to the laboratory for dissection. The superficial digital flexor (SDF) tendon, an energy storing tendon, and the common digital extensor (CDE) tendon, a positional tendon, were dissected from each forelimb using a scalpel. A 3-cm-long piece was taken from each tendon, centrally located between bone and muscle attachments. Each tendon sample was placed in a 50 mL Falcon tube filled with 0.15 M phosphate buffered saline (PBS) solution plus 1% amphotericin B (0.25 mg/mL) and 1% penicillin-streptomycin (100 units/mL pen, 0.1 mg/mL strep). Each tendon then underwent decellularization treatment following the procedure of Ariganello *et al*.^47^. While specifically designed to remove cellular material from connective tissue, the utility of the treatment for this study was in allowing individual collagen fibrils to be easily extracted from the tendon samples. Briefly, the tendon samples underwent three 12 h washes at 4 °C in hypotonic solution, followed by two 12 h washes at 4 °C in 1% Triton X-100 solution. The samples were then incubated in a DNAse/RNAse solution at 37 °C for 1 h, washed at room temperature for 12 h in sterile 1% Triton X-100 solution twice, and rinsed using two 30 min washes and then a final 46 h wash in sterile PBS with 1% amphotericin B and 1% penicillin-streptomycin. This treatment procedure has been previously applied to tendons, and has been found not to alter collagen fibril ultrastructure or fibril response to mechanical overload^48^.

### Collagen fibril extraction & testing preparation

To extract single collagen fibrils, each 3-cm-long piece of decellularized tendon was placed in a plastic dish containing 20 mL of room temperature PBS. Using a razorblade, a central rectangular plug was cut from each tendon, which was free of epitenon. The rectangular plug was sliced longitudinally using the razorblade, and then splayed open along the cut using metal tweezers. The exposed interior of the plug was scraped with the razorblade for 10 min, which released fibrils into the surrounding liquid. While the goal of this procedure was to extract the longitudinally oriented collagen fibrils that form the tendon’s bulk substance, it is possible that some fibrils from endotenon were also extracted. Using a plastic pipette, the resulting collagen fibril/PBS mixture was distributed in 1 mL aliquots amongst 10 glass sample dishes. The dishes were then put on a linear shaker table operating at 1 Hz for 30 minutes to allow the fibrils to settle and adhere to the glass substrate. The linear shaking motion was used to encourage parallel alignment between the settled fibrils. After 30 minutes, each dish was successively rinsed three times under agitation with 3 mL of Nanopure water to remove any salts, dried under compressed nitrogen, and stored in a desiccating chamber for 24 h. The dishes containing dried fibrils were then examined using darkfield microscopy, and straight, single fibrils ≥ 100 μm were identified. Under microscopic guidance, a fine-tipped glass rod controlled via a 3D hydraulic micromanipulator (Siskiyou Corp, USA) was used to place two parallel strips of 5 min epoxy (LePage, USA) spaced by approximately 50 μm across each selected fibril. The ~50 μm length (mean ± SD: 55.9 ± 8.2 μm) of each fibril between the glue strips was used for tensile testing; the portion of the same fibril outside of the glue strips served as an unloaded control for the subsequent structural analyses (Fig. 1). After selecting and preparing multiple fibrils on each glass dish in this manner, the dishes were returned to a desiccator for a minimum of 24 hours (maximum 72 hours) prior to rehydration and tensile testing. A total of 38 fibrils were prepared in this manner: 21 fibrils from positional CDE tendons, and 17 fibrils from energy storing SDF tendons.

### Pre-test AFM imaging

AFM imaging and tensile testing of the single collagen fibrils extracted from CDE and SDF tendons (n = 21 CDE fibrils, n = 17 SDF fibrils) was performed using an atomic force microscope (Bioscope Catalyst, Bruker, USA) mounted on an inverted optical microscope (IX71, Olympus, USA) outfitted with a 100X, 1.3 NA objective (Olympus, USA) and a Grasshopper CCD camera (Point Grey, Canada). After removal from the desiccator following gluing and prior to rehydration, the AFM was used to image a 500 nm length of each fibril to be tested. This was done using a ScanAsyst fluid + probe (nominal spring constant 0.7 N/m) operated in peak force quantitative nanomechanical mapping mode. The images had a pixel size of 8 nm, the tip velocity was 1200 μm/s, the raster scan frequency was 0.5 Hz, and the peak force setpoint was 10 nN. Using Nanoscope Analysis software, the average cross-sectional profile of each fibril to be tested was determined and then integrated to obtain its dry cross-sectional area (CSA). Approximating the dry fibrils as cylinders, the average diameters of the tested positional CDE and energy storing SDF fibrils were 247 ± 29 nm and 144 ± 16 nm, respectively.

### Tensile testing

After dry imaging of the fibrils to be tested was complete, each glass sample dish was filled with 3 mL of room temperature PBS and left for 1 h to rehydrate the fibrils contained within. Tensile testing of the rehydrated fibrils was conducted using a calibrated Bruker Tap525A probe (average vertical spring constant 150 N/m, average lateral spring constant 3800 N/m, see Kreplak *et al*.^49^ for details). Sample dishes were oriented on the AFM such that the longitudinal axis of the fibril to be tested was parallel to the axis of the AFM cantilever, which was fixed. To perform a tensile test, the AFM probe was brought into contact with the glass close to the midpoint of the selected fibril segment with an applied normal force of 15 μN. The AFM stage was moved at a constant velocity of 1 μm/s perpendicular to the fibril axis while the lateral force on the AFM probe was recorded at 500 Hz and a video of the test was recorded at 20 fps. Great care was taken to always have the entire isolated fibril segment and part of the two restraining glue strips in view during the entire test to check for fibril slippage. During a typical test, the probe first bent the fibril gently until it reached a triangular shape. Once in this geometry, and assuming the probe is exactly at the midpoint of the fibril segment, the strain *ε* and tension *T* are non-linear functions of time and can be computed as follows^50^:

1 $$\varepsilon =\sqrt{1+4{(\frac{vt}{{L}_{o}})}^{2}}-1$$

2 $$T=\,{F}_{Lateral}\frac{\sqrt{{L}_{o}^{2}+4{(vt)}^{2}}}{4vt}$$

with *v* the stage velocity, *L*_0_ the initial, undeformed segment length, *t* the time, and *F*_*Lateral*_ the lateral force on the AFM probe. The raw force-displacement data (collected at 500 Hz) was processed to subtract the frictional force between AFM tip and glass substrate, and was then smoothed by averaging every five points (Fig. S3). Engineering stress *σ* was simply computed as tension *T* divided by the dry CSA of the fibril. With the origin of time taken when the AFM probe first made contact with a fibril, stress and strain were calculated starting from when fibrils first reached a triangular shape, with both time points determined via video. It is important to note that when the test segment of a fibril reached a triangular shape and the first stress-strain data could be calculated, the fibril was already under notable stress and strain. The portion of each stress-strain curve prior to this was approximated by a linear line connecting this first calculated data point back to the origin (the zero stress, zero strain state of the fibril before contract by the AFM tip). Four mechanical parameters were measured to describe each stress-strain curve: rupture strain, rupture stress, toughness, and high strain elastic modulus. Rupture strain and rupture stress were measured from the last data point prior to the stress abruptly falling to zero. Toughness was the integral of the stress-strain curve, evaluated from 0% strain to the rupture strain value. High strain elastic modulus was calculated as the slope for the stress-strain curve for the last 10% strain preceding rupture.

### Post-rupture AFM imaging

All fibrils underwent post-rupture AFM imaging. After rupturing all fibrils on a given dish, the dish was removed from the AFM stage, rinsed three times using Nanopure water to remove any salt, dried under compressed nitrogen, and stored in a desiccating chamber. The dehydrated, ruptured fibrils were then imaged using AFM to characterize morphological changes. These images were typically between 30–40 μm wide, and taken with a 0.125 Hz raster scan frequency. Other imaging parameters were the same as those used during the pre-test AFM imaging for CSA measurement. In addition to qualitative assessments, the images were used to quantify the number of discrete damage sites present along the length of each ruptured fibril, and quantify the degree of each fibril’s radial structural disruption by subtracting the post-rupture fibril core height from that measured prior to rupture.

### Second harmonic generation imaging

The rupture-induced structure of 16 CDE fibrils and 14 SDF fibrils were further explored via second harmonic generation imaging (SHG), performed using a custom-built laser scanning microscope described previously^51,52^. Samples were probed with 150 fs linearly polarized laser pulses at 810 nm wavelength, generated with a titanium:sapphire oscillator^51^. The scattered SHG light at 405 nm wavelength was collected in the forward and backward directions with respect to the excitation, and was isolated using two FF01–720/SP-25 lowpass filters (Semrock, USA) that rejected the excitation light, and one FF01–405/10–25 bandpass filter (Semrock, USA) that selected the SHG wavelength and rejected the room light. Images were acquired point by point by scanning the focused excitation spot across the sample with a galvanic mirror (as described by Houle *et al*.^51^). A 40X, NA 1.1, water dipping objective (UAPO W3/340, Olympus, Japan) was used, yielding 400 nm spatial resolution. The pixel size was selected to be 200 nm, in order to oversample the fibril structure. The pixel dwell time was 20 µs, leading to typically 5 s acquisition time for a single 500 × 500 pixels image. Average laser power at the objective focus was set to approximately 15 mW using a half-waveplate and a Glan Thompson polarizer. For each ruptured fibril, the linear polarization of the excitation laser was rotated through [0°−170°] in 10° increments, yielding a pol-stack of 18 images. The maximum intensity value at each pixel, selected from amongst the 18 images in the pol-stack, was used to generate a single polarization-corrected maximum intensity map for each fibril. The field of view of the pol-stacks included both the ruptured and unloaded control portions of each fibril, and an average intensity value was measured for both portions. A ratio of ruptured/unruptured SHG signal was calculated for each fibril using these measurements. This analysis was performed for both forward and backward scattering.

The SHG response of a collagen fibril, upon excitation by the incident laser beam, is characterized by the second-order non-linear susceptibility tensor (χ^(2)^). Assuming that the fibrils have a cylindrical symmetry (C_6*v*_) and that the Kleinman symmetry is valid in collagen^53,54^, the non-linear susceptibility of fibril lying along the x-axis only exhibits two independent tensor components: χ^(2)^_xxx_ and χ^(2)^_xyy_. Therefore, the SHG response of the fibril to the excitation beam, propagating along the z-axis, with a linear polarization within the xy-plane is described by:

3 $${I}_{2\omega }\propto {[{{\rm{\rho }}\cos }^{2}(\phi -\alpha )+{\sin }^{2}(\phi -\alpha )]}^{2}+{\sin }^{2}(2(\phi -\alpha ))$$

where α and *φ* are the angles of the polarization and the fibril with respect to the x-axis respectively and ρ is the ratio of the two independent tensor component (χ^(2)^_xxx_/χ^(2)^_xyy_). This ratio reflects the anisotropy of the nonlinear response and provides insight into the orientation disorder of the collagen triple helices within the focal volume. To extract the anisotropy parameter from the data, we used the fast-Fourier transform (FFT)-based approach reported by Amat-Roldan *et al*.^55^. To that end, equation (3) can be expressed as a sum of Fourier component:

4 $${I}_{2\omega }\propto {a}_{o}+{a}_{2}\,\cos (2(\phi -\alpha ))+{a}_{4}\,\cos (4(\phi -\alpha ))$$

where the three parameters a_0_, a_2_ and a_4_ now contain all the information relative to the tensor element and thus to the anisotropy. As previously demonstrated, the anisotropy parameters can now be determined using:

5 $$\rho =\sqrt{\frac{{a}_{4}+{a}_{2}+{a}_{0}}{{a}_{4}-{a}_{2}+{a}_{0}}}$$

### Fluorescence-based detection of denatured collagen

To assess whether rupture led to differences in the denaturation (uncoiling) of collagen molecules in CDE and SDF fibrils, a fluorescent collagen hybridizing peptide was employed, which binds to denatured collagen, but not intact triple helical collagen^40^. Labelling and imaging of both the unloaded and ruptured portions of 11 CDE fibrils and 15 SDF fibrils was conducted.

Fibrils were first rehydrated for 1 h at room temperature by adding 3 mL of ultrapure water to their respective dishes. A 10 μM solution of fluorescein conjugated collagen hybridizing peptide (CHP) (Echelon Biosciences Inc., Salt Lake City, UT) was prepared, heated for at 80 °C for 5 min, and then immersed in an ice bath for 15 sec. The water from the sample dishes was then replaced with the CHP solution. Samples were incubated with the CHP solution for 12 h at 4 °C. After incubation, each dish underwent three 5 min rinses with ultrapure water to remove free CHP. Dishes were then dried under argon.

Fluorescent imaging was conducted using a Zeiss LSM 710 upright confocal microscope. An argon ion laser was used to excite the samples at 488 nm. Micrographs were acquired and processed with ZEN 2009 Microscope and Imaging Software. Samples were imaged with a 40 × oil immersion lens with digital zooms of 2.0 × and 5.5 × . In order to isolate selected fibrils for scanning, a region of interest (ROI) function was used. Subsequently, laser scanning and bright field micrographs were acquired with a 2048 × 2048 pixel resolution. Micrographs were acquired with a pixel dwell of 6.3µs and averaged across 2 scans per line.

### Statistics

Numerical data are presented as mean ± standard deviation. Statistical analyses were conducted using JMP software (version 13.0, SAS Institute Inc., Cary, NC). Data were checked for normality using Shapiro-Wilk tests, and homogeneity of variances were checked using Lavene’s test.

Mechanical properties (Fig. 3), number of plastic damage sites (Fig. 6A), fractional change in fibril core height (Fig. 6B), and fractional change in maximum forward SHG (Fig. 6C) all contained non-normally distributed groups. These data were therefore rank transformed before being analyzed using two-way ANOVAs with the factors tendon type and animal of origin. In addition to tendon type being a significant main effect, for rupture strain and the number of plastic damage sites, a significant interaction effect existed, and for fractional change in fibril core height animal of origin was also a significant main effect. These data are therefore shown separated by both tendon type and animal. Following two-way ANOVA, differences between tendon types were compared using Wilcoxon rank sum tests.

For both the mean and FWHM SHG anisotropy data (Fig. 7C,D), paired differences between the control and ruptured portion of each fibril were first analyzed using a two-way ANOVA with the factors tendon type and animal of origin. Only the main effect of tendon type was significant for each. The data for both animals was therefore combined, and the effect of rupture for each tendon type assessed using matched-pair t-tests.

## Electronic supplementary material

Supplementary information
