# Supplementary material for: In tendons, differing physiological requirements lead to functionally distinct nanostructures
Source: Sci Rep. 2018 Mar 13;8:4409. doi: 10.1038/s41598-018-22741-8 (PMC5849720; doi:10.1038/s41598-018-22741-8)
Supplement: Supplementary file 1 — Supplementary information [file 41598_2018_22741_MOESM1_ESM.pdf]

Supplementary information for:

In tendons, differing physiological requirements lead to functionally distinct nanostructures

Andrew S Quigley[1], Stéphane Bancelin[2], Dylan Deska-Gauthier[3], François Légaré[2], Laurent Kreplak[1,4*], Samuel P Veres[4,5*]

[1]Department of Physics and Atmospheric Science, Dalhousie University, Halifax, Canada.

[2]Institut National de la Recherche Scientifique, Centre Énergie, Matériaux, Télécommunication, Varennes, Canada.

[3]Department of Medical Neuroscience, Dalhousie University, Halifax, Canada.

[4]School of Biomedical Engineering, Dalhousie University, Halifax, Canada.

[5]Division of Engineering, Saint Mary's University, Halifax, Canada.

*Corresponding authors. Email: sam.veres@smu.ca; kreplak@dal.ca

Collagen Fibrils from Positional CDE Tendons

Positional fibrils with a two phase stress-strain response (19/21 fibrils):

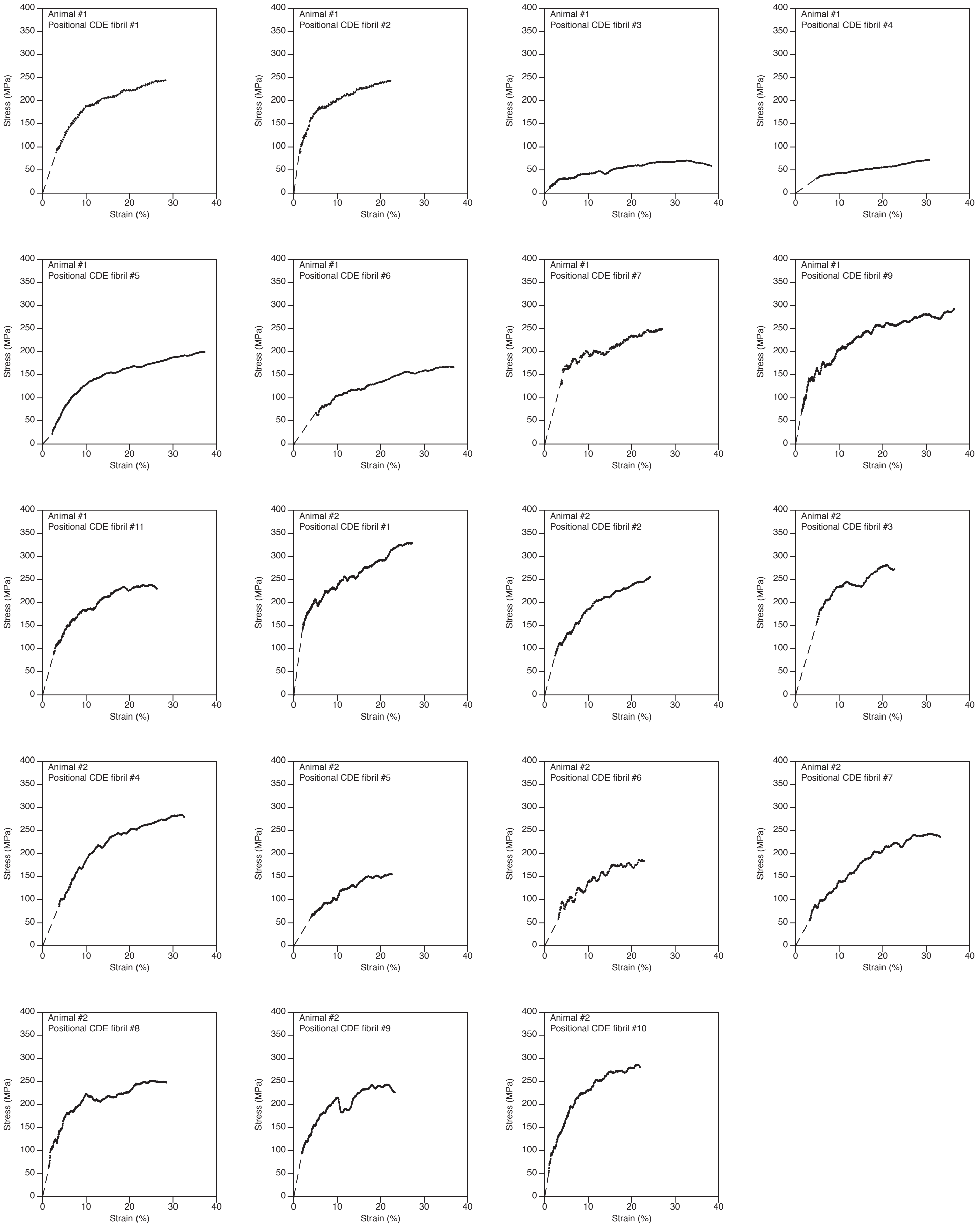

Positional fibrils with a three phase stress-strain response (2/21 fibrils):

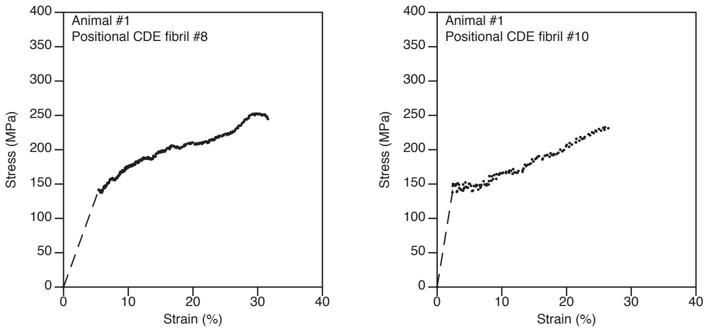

Figure S1. Stress-strain curves for collagen fibrils extracted from bovine forelimb CDE tendons and loaded to rupture. Of the 21 fibrils tested, 19 displayed a two-phase load-elongation response where a marked decrease in stiffness occurred at approximately 10% strain and persisted until rupture.

Collagen Fibrils from Energy Storing SDF Tendons

Energy storing fibrils with a three phase stress-strain response (12/17 fibrils):

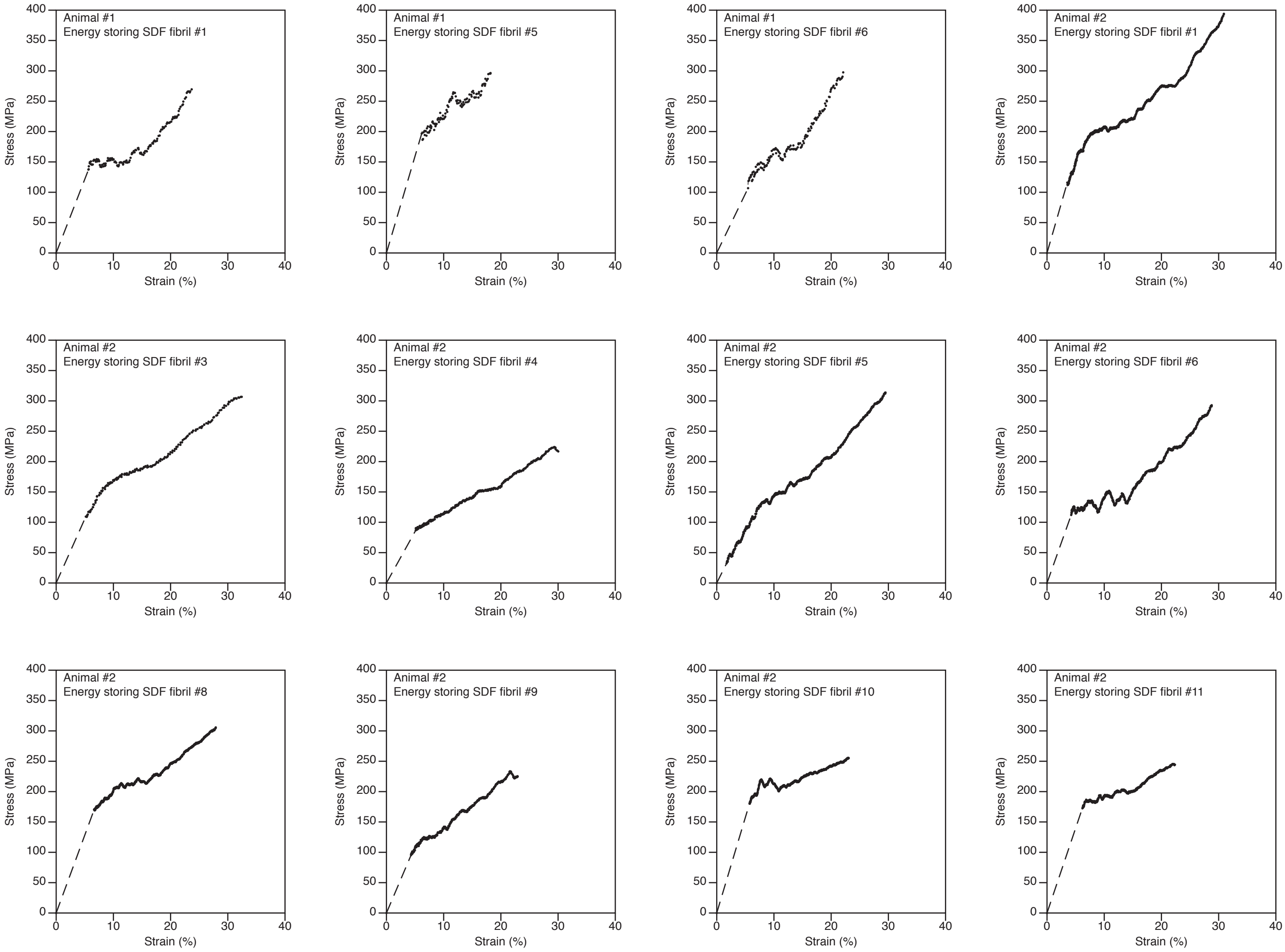

Energy storing fibrils with a two phase stress-strain response (5/17 fibrils):

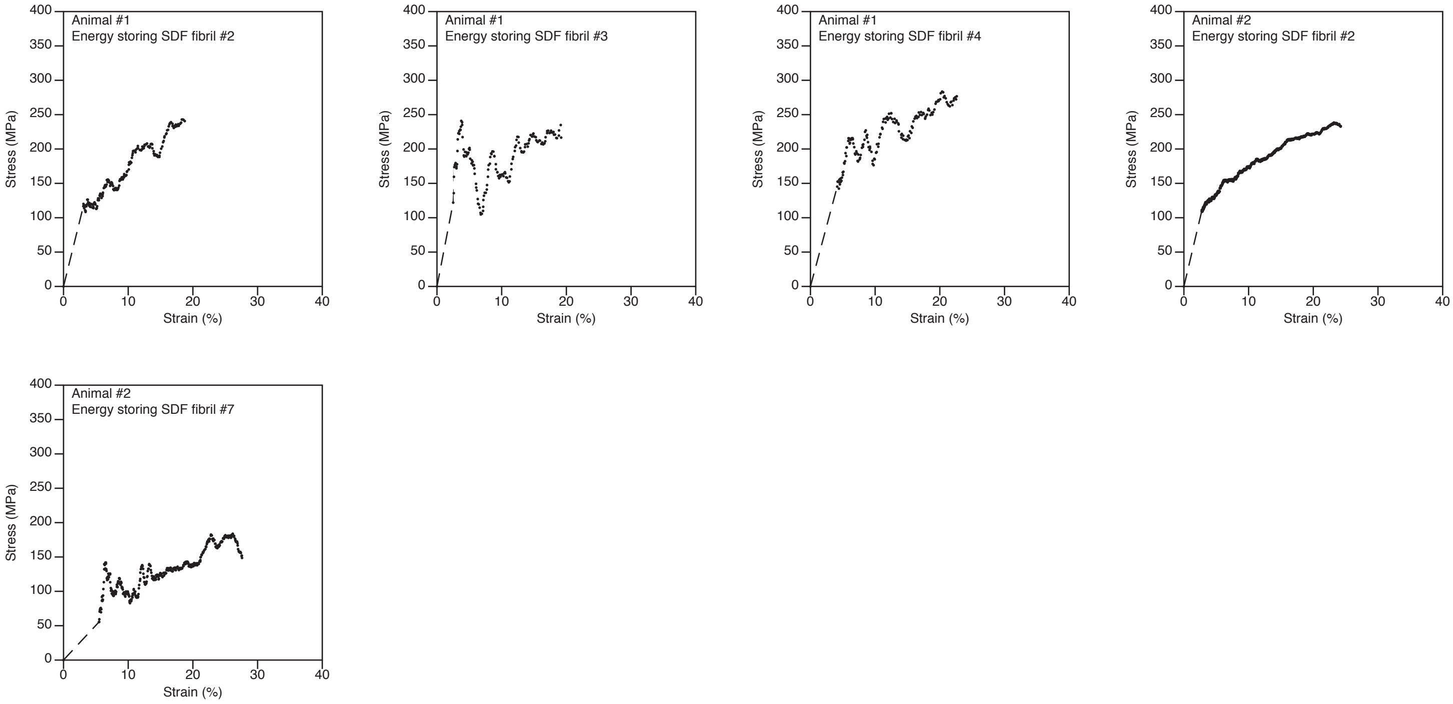

Figure S2. Stress-strain curves for collagen fibrils extracted from bovine forelimb SDF tendons and loaded to rupture. Of the 17 fibrils tested, 12 displayed a three-phase load-elongation response where a marked decrease in stiffness occurred at approximately 10% strain, followed by a marked increase in stiffness at approximately 15% strain, which persisted until rupture.

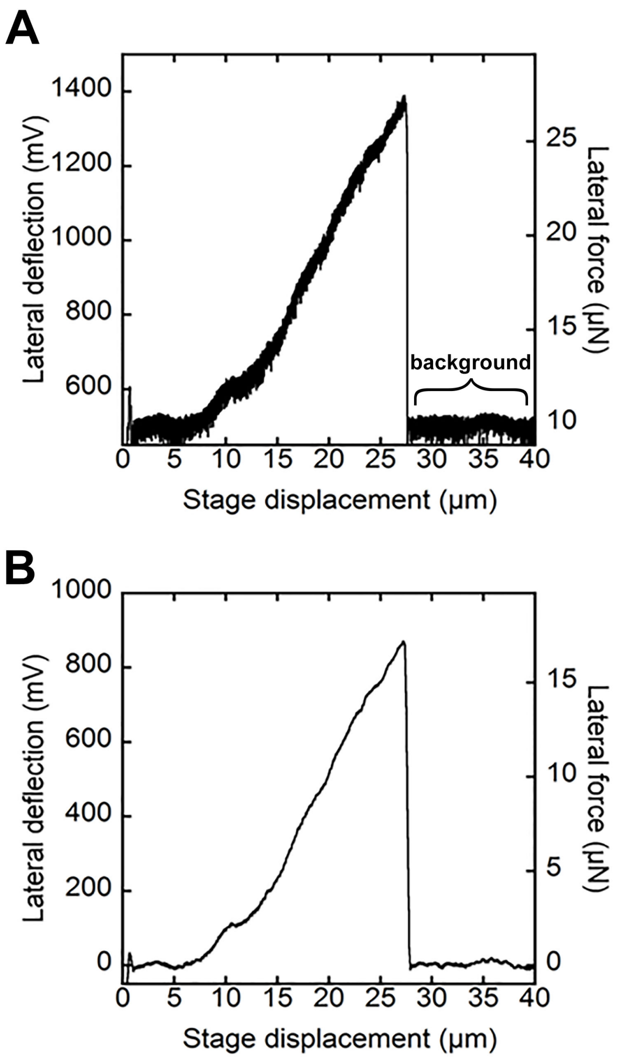

Figure S3. **A**: Raw force-displacement curve for a representative positional CDE fibril. Force-displacement data was collected at 500 Hz. The portion of the force-displacement curve following fibril rupture, labelled "background", shows the frictional force between AFM tip and glass substrate. The average value of the frictional force was subtracted from the force data during processing. **B**: The force-displacement curve based on the data shown in **A** after removal of the background frictional force and averaging over five points to smooth the data.
